# An Intra-Cycle Optimal Control Framework for Ventricular Assist Devices Based on Atrioventricular Plane Displacement Modeling

**DOI:** 10.1007/s10439-021-02848-2

**Published:** 2021-09-21

**Authors:** Clemens Zeile, Thomas Rauwolf, Alexander Schmeisser, Jeremi Kaj Mizerski, Rüdiger C. Braun-Dullaeus, Sebastian Sager

**Affiliations:** 1 The Institute of Mathematical Optimization, Otto-v.-Guericke-Universität, Universitätspl. 2, 39106 Magdeburg, Germany; 2 The Department of Heart Surgery, SPSW im. pap. Jana Pawła II, Aleja Jana Pawła II 10, 22-400 Zamość, Poland; 3 The Department of Cardiology, Otto-v.-Guericke-Universität, Leipziger Str. 44, 39120 Magdeburg, Germany

**Keywords:** Heart failure, Left ventricular assist device, Optimal control, Switched systems

## Abstract

A promising treatment for congestive heart failure is the implementation of a left ventricular assist device (LVAD) that works as a mechanical pump. Modern LVADs work with adjustable constant rotor speed and provide therefore continuous blood flow; however, recently undertaken efforts try to mimic pulsatile blood flow by oscillating the pump speed. This work proposes an algorithmic framework to construct and evaluate optimal pump speed policies with respect to generic objectives. We use a model that captures the atrioventricular plane displacement, which is a physiological indicator for heart failure. We employ mathematical optimization to adapt this model to patient specific data and to find optimal pump speed policies with respect to ventricular unloading and aortic valve opening. To this end, we reformulate the cardiovascular dynamics into a switched system and thereby reduce nonlinearities. We consider system switches that stem from varying the constant pump speed and that are state dependent such as valve opening or closing. As a proof of concept study, we personalize the model to a selected patient with respect to ventricular pressure. The model fitting results in a root-mean-square deviation of about 6 mmHg. The optimization that considers aortic valve opening and ventricular unloading results in speed modulation akin to counterpulsation. These in silico findings demonstrate the potential of personalized hemodynamical optimization for the LVAD therapy.

**Supplementary Information:**

The online version contains supplementary material available at 10.1007/s10439-021-02848-2.

## Introduction

Left ventricular assist devices (LVADs) provide mechanical circulatory blood support and have become a well-established and successful therapy for end-stage heart failure patients with estimated more than 5000 implanted pumps annually worldwide.^16,28^ The role of the heart assist devices is growing in recent years since there are major improvements in the long term treatment.^36^ Contemporary LVADs implement rotary continuous blood flow and are internally implanted in contrast to pulsatile and extracorporeal pumps, which represent the original LVAD design, but which are bigger, less durable, and more invasive than their continuous flow counterpart.^54^

These either axial- or centrifugal-flow pumps were originally designed to apply a fixed constant rotary speed. However, there is evidence that this lack of pulsatile flow can cause numerous adverse effects that include gastrointestinal bleeding,^17^ reduced end-organ function,^45^ aortic valve thrombosis and *de novo* aortic insufficiency.^18^ To this end, the latest generation of devices features a pulsatile mode in addition to the constant speed option that oscillates the motor rotation speed periodically for a short period of time before returning to the constant speed operation. Examples of these devices and modes are HeartMate 3^TM^ with the *Pulse mode*, HeartWare HVAD^TM^ with the *Lavare Cycle* and EXCOR/INCOR^TM^.^29^ For further details on the devices, the medical background, therapy planning and prognosis we refer to the reviews.^11,27,32,37^

### Related Work

A vast amount of preclinical models for evaluating and testing LVADs *via* pump speed modulation have been proposed. Amacher *et al*.^4^ reviewed a range of studies^47,52,60,61^ where a preselected constant, sine or square wave speed profile is assumed. Chosen parameters were adjusted for amplitude and phase shift to analyzing the effect on relevant physiological quantities. Specifically, high-speed pumping during ventricular contraction, also denoted as copulsative mode, was found to be beneficial in terms of pulsatility in the systemic arterial circulation. Counterpulsative pumping, i.e., low-speed pumping during the ventricular contraction, enhanced left ventricle (LV) unloading.^44^

A preselected speed profile does not adjust to dynamic changes in the state of the cardiovascular system. For this reason, control strategies for the blood pumps were developed that take into account different physiological objectives and which were classified in the review of Bozkurt.^12^ Physiological control following the Frank–Starling mechanism by pumping preload dependent has been proposed in References 6, 21, 50. Control algorithms that aim for unloading the LV were elaborated in References 13, 43. Speed regulation algorithms for generating sufficient perfusion and detecting ventricular suction^10,20^ or pulmonary oxygen gas exchange tracking^30^ are other goals, and, finally, multi-objective variants exist.^46^

Due to the increased necessity of LVADs for clinical use, a wide range of different methods from control engineering has been proposed, such as adaptive,^42,65^ robust,^48^ model predictive,^1^ fuzzy logic,^14^ proportional integral derivative,^25^ sliding mode,^8^ and iterative learning control.^34^ We refer to Reference 2 for a detailed review and for a discussion on the applicability of these methods in clinical practice.

### Contributions

This case study follows an optimal control approach since it offers a flexible framework to include and combine multiple objective and constraint functions. So far, optimal control studies based on cardiovascular system modeling appear to be very limited in the context of ventricular assist devices. Reference 24 investigated the use of LVADs for preload manipulation maneuvers in animal trials. We build on Reference 3, where the continuous pump speed profile is found with an optimal control algorithm based on a lumped cardiovascular system model and compared with both a constant and a sinusoidal-speed profile. In contrast, we do only numerical simulation and no verification with a mock circulation system. Our idea is to consider the cardiovascular dynamics as a system that *switches* between different phases in a single cardiac cycle, e.g., valve opening or closing, in which different dynamics apply. We use solving techniques tailored for *switched systems* to reduce the underlying system nonlinearities and leverage the computations. Within this framework, we present a novel algorithm to calculate optimal piecewise constant (pwc) pump speed modulation following the above-mentioned pulsatility modes for modern devices and concerning ventricular unloading and opening of the aortic valve. For comparison, we compute the optimal continuous and constant speed profiles. In this study, we consider a single cardiac cycle to control intra-cycle unloading, but claim that our approach can be extended to longer time horizons. Furthermore, we consider adapting model parameters to patient-specific data with a nonlinear regression objective function to deal with a personalized model.

Another fundamental difference between our approach to Reference 3 and all other model-based approaches lies in the used model. Instead of applying a time-varying elastance function to represent the pressure–volume relationship in heart chambers, we base our model on the contribution of the longitudinal atrioventricular plane displacement (AVPD) to ventricular pumping, which is novel in the LVAD context. It has been established that the atrioventricular plane (AVP) behaves like a piston unit by moving back and forth in the base-apex direction, creating reciprocal volume changes between atria and ventricles.^39^ Also, there is strong evidence that the magnitude of AVPD is a reliable index for heart failure diagnosis.^64^

Elastance functions are used in many LVAD studies with realistic results (e.g. Reference 3), but it is controversial whether they accurately simulate the dynamic interaction between the LV and an assist device.^15,62^ As an alternative, we reuse and extend an AVPD model introduced in Reference 40 and altered to the switched systems setting in Reference 31. Other alternatives for replacing the elastance model are myofiber, or sarcomere mechanics approaches^35^ as in the CircAdapt model,^7,38^ though a great number of discontinuities and nonlinear equations limit their applicability to (gradient-based) optimization and control techniques. The presented approach is clinically applicable since the AVP motion is relatively easy to measure *via* noninvasive echocardiography.

The outline of this article is the following: We describe the cardiovascular and LVAD system model in  “Cardiovascular System and LVAD Modeling” section, before we define constraints in  “Physiological Assumptions and Constraints” section, the clinical data and model personalization in “Clinical Data and Model Personalization” section. Afterwards, we formulate the optimal control problem (OCP) in “Optimal Control Problem Formulation” section and we define an algorithmic approach to solve it in “Algorithmic Approach” section. We present simulation results in “Results” section and discuss the realistic and algorithmic setting together with limitations in “Discussion” section. We wrap up the article with conclusions in “Concluding Remarks” section.

## Materials and Methods

### Cardiovascular System and LVAD Modeling

This study uses a lumped model of the cardiovascular system based on the representation of the left heart, neglecting the right heart and the pulmonary system. We combine the AVPD model as proposed and validated in Reference 31 with an axial HeartMate II^TM^ pump LVAD model that has been validated in Reference 53, but claim that our approach is generic, i.e. another LVAD model could also be used. The proposed model consists of nine ODEs for the pressure *P*(*t*) (mmHg) of left atrium (LA), LV, aorta (A), systemic artery (S), and venous system (V), the flow *Q*(*t*) (mL/s) in the A and in the LVAD as well as the velocity *v*(*t*) (cm/s) of the A and its position *s*(*t*) (cm), where (*t*) denotes the time dependency. The cardiovascular system can be steered with the continuous control *u*(*t*) that represents the rotary pump speed. The ODE system reads for $t\in [t_{0}, t_{\text {f}}]\subset \mathbb {R}$:

1a $$\begin{aligned} {\dot{P}}_{\text {LA}}(t)&= \frac{P_{\text {V}}(t)-P_{\text {LA}}(t)}{C_{\text {LA}}R_{\text {V}}}- \frac{Q_{\text {MV}}(t) - A_{\text {LA}} v(t)}{C_{\text {LA}}}, \end{aligned}$$

1b $$\begin{aligned} {\dot{P}}_{\text {LV}}(t)&= \frac{(1+ k_{\text {RAD}})A_{\text {LV}} v(t)}{C_{\text {LV}}} + \frac{Q_{\text {MV}}(t)- Q_{\text {AoV}}(t)-Q_{\text {LVAD}}(t)}{C_{\text {LV}}}, \end{aligned}$$

1c $$\begin{aligned} {\dot{P}}_{\text {A}}(t)&= \frac{ Q_{\text {AoV}}(t) + Q_{\text {LVAD}}(t)- Q_{\text {A}}(t) }{C_{\text {A}}}, \end{aligned}$$

1d $$\begin{aligned} {\dot{P}}_{\text {S}}(t)&= \frac{P_{\text {V}}(t)-P_{\text {S}}(t)}{C_{\text {S}} R_{\text {S}}}+ \frac{Q_{\text {A}}(t)}{C_{\text {S}}} , \end{aligned}$$

1e $$\begin{aligned} {\dot{P}}_{\text {V}}(t)&= \frac{P_{\text {S}}(t)-P_{\text {V}}(t)}{C_{\text {V}} R_{\text {S}}} + \frac{P_{\text {LA}}(t)-P_{\text {V}}(t)}{C_{\text {V}} R_{\text {V}}}, \end{aligned}$$

1f $$\begin{aligned} {\dot{Q}}_{\text {A}}(t)&= \frac{ P_{\text {A}}(t) - P_{\text {S}}(t) - R_{\text {C}} Q_{\text {A}}(t)}{L_{\text {S}}}, \end{aligned}$$

1g $$\begin{aligned} {\dot{Q}}_{\text {LVAD}}(t)&= \frac{ P_{\text {LV}}(t) - P_{\text {A}}(t) - R_{\text {LVAD}} Q_{\text {LVAD}}(t) - \beta u(t)^{2} }{L_{\text {LVAD}}}, \end{aligned}$$

1h $$\begin{aligned} {\dot{v}}(t)&= \frac{-R_{\text {AVP}} v(t) - A_{\text {LV}} P_{\text {LV}}(t) + A_{\text {LA}}P_{\text {LA}}(t) + F_{\text {C}}(t) }{L_{\text {AVP}}}, \end{aligned}$$

1i $$\begin{aligned} {\dot{s}}(t)&= v(t), \end{aligned}$$

where the default parameter values for the compliances *C*, resistances *R*, and inertances *L* are given in the Supplemental Material 1. The model uses the valve flows^1^ defined by

2a $$\begin{aligned} Q_{\text {MV}}(t)&= \left\{ \begin{array}{ll} \frac{P_{\text {LA}}(t)- P_{\text {LV}}(t)}{R_{\text {M}}},&{} \quad \text {if } P_{\text {LA}}(t) > P_{\text {LV}}(t), \\ 0, &{} \quad \text {else.} \end{array} \right. \end{aligned}$$

2b $$\begin{aligned} Q_{\text {AoV}}(t)&= \left\{ \begin{array}{ll} \frac{P_{\text {LV}}(t)- P_{\text {A}}(t)}{R_{\text {AoV}}},&{} \quad \text {if } P_{\text {LV}}(t) > P_{\text {A}}(t), \\ 0, &{} \quad \text {else.} \end{array} \right. \end{aligned}$$

The ventricular (AV) plane contraction force is assumed to be a pwc function in the following sense

3 $$\begin{aligned} F_{\text {C}}(t) = \left\{ \begin{array}{ll} F_{\text {AC}},&{} \quad \text {during atrial contraction} , \\ F_{\text {VC}},&{} \quad \text {during ventricular contraction} , \\ 0, &{} \quad \text {else.} \end{array} \right. \end{aligned}$$

We specify in “Algorithmic Approach” section how these contraction phases are mathematically defined and skip their formal introduction here.

**Figure 1:**
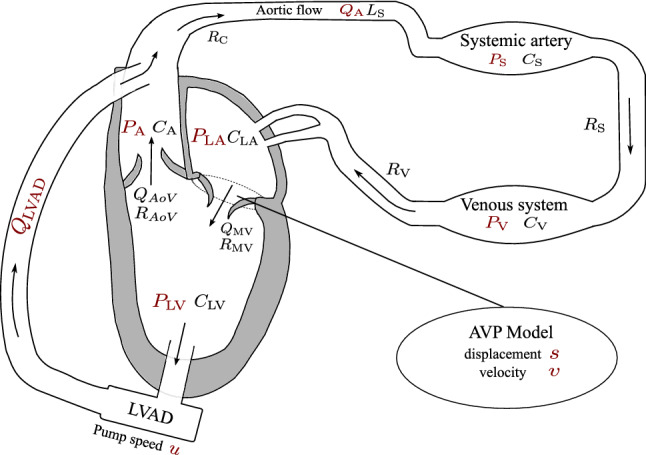
Illustration of the simplified model of the left heart, the circulatory system, and the LVAD. Differential states and the pump speed control *u*(*t*) are depicted in red. The cyclic flow is indicated by the arrows. The model consists of five compartments for the left atrium (LA), left ventricle (LV), aorta (A), systemic artery, and venous system, represented with the pressure functions *P*(*t*). These variables interact with the flows *Q*(*t*) in the LVAD and the aorta, while the AV interaction is modeled by the velocity *v*(*t*) and position *s*(*t*) of the atrioventricular plane displacement (AVPD). Compliance, resistance and inertance parameters *C*, *R*, *L* are depicted next to the corresponding compartment.

Figure 1 gives a schematic overview of the lumped model of the heart and the circulatory system. In the following, we group the differential states into the vector

4 $$\begin{aligned} \mathbf {x}=[P_{\text {LA}}, P_{\text {LV}}, P_{\text {A}}, P_{\text {S}}, P_{\text {V}}, Q_{\text {A}}, Q_{\text {LVAD}}, v, s]^{\top }\end{aligned}$$

and write the dynamical system (1a)–(1i) as

5 $$\begin{aligned} \dot{\mathbf {x}}(t) = \mathbf {f}(\mathbf {x}(t),u(t)), \quad \text { for } t\in [t_{0},t_{\text {f}}]. \end{aligned}$$

**Figure 2:**
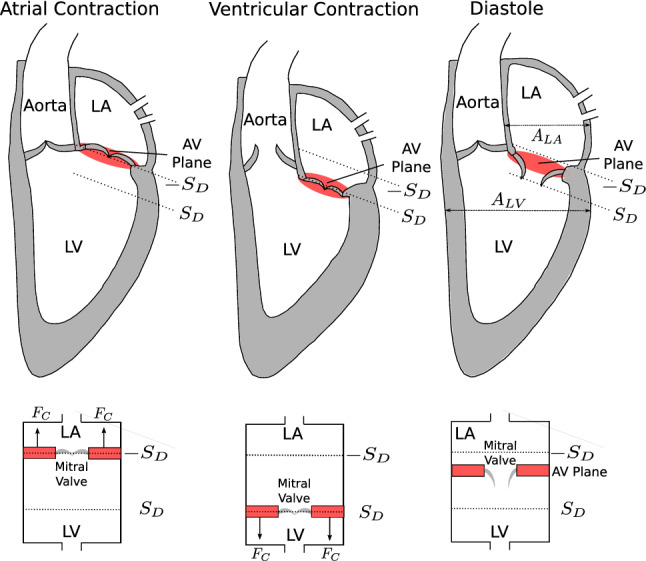
Illustration of the atrioventricular plane displacement concept for the left heart. The atrioventricular plane moves forth and back between $-S_{\text {D}}$ and $S_{\text {D}}$, pulled by the contraction force $F_{\text {C}}$ resulting in blood redistribution from LA to the LV to the Aorta. This behavior resembles a piston pump, as depicted at the bottom, where $-S_{\text {D}}$ and $S_{\text {D}}$ mark the longitudinal displacement into basal and apical direction respectively.

Figure 2 illustrates the AVPD model, where the AVP refers to the separating tissue between LV and LA that surrounds the mitral valve. During atrial contraction, the force $F_{\text {C}}$ pulls the AVP towards the base and redistributes blood from the LA to the LV *via* the mitral valve. When it reaches the switching threshold $-S_{\text {D}}$, the contraction force $F_{\text {C}}$ starts to work in the opposite direction, which represents ventricular contraction. In this way, the AVPD leverages longitudinal pumping that results in ejection of blood to the Aorta. The ventricular contraction stops as soon as the AVP reaches the threshold $S_{\text {D}}$. A relaxation phase follows where $F_{\text {C}}$ equals zero and the AVP moves slowly to its original position. This longitudinal pumping is well described by a piston unit concept, where the piston is placed between LA and LV with constant cross-sections $A_{\text {LA}}$ and $A_{\text {LV}}$ respectively. We illustrate this piston representation at the bottom of Fig. 2. The AVP model assumes that longitudinal pumping is supported by the radial squeezing of LV walls. Many morphological changes accompany end-stage heart failure influencing AVP motion. The AVP model can be adapted to this situation since the parameter $S_{\text {D}}$ captures the AVPD.

### Physiological Assumptions and Constraints

This study makes a series of assumptions, which are explained here. We list the applied constraint parameter values in Supplemental Material 2.

#### Dilated Left Heart Failure

The proposed model is adapted in order to represent a typical LVAD patient candidate’s heart situation.^27^ This includes modeling left-sided heart failure with decreased cardiac output and dilated cardiomyopathy^2^ with enlarged LA and LV. To this end, we modify certain model parameters, including an increased compliance and increased cross-sectional area of LV as described in more detail in Supplemental Material 1. In addition, further parameters can be adapted to a specific patient, as explained in “Clinical data and Model Personalization” section.

#### Steady-State Situation

We assume the cardiovascular and circulatory system is in *steady-state*, in the sense of

- there are no rapid or major changes of cardiac output and the heart cycle length, meaning these two quantities hardly change over different cardiac cycles,
- the system has already adapted to the LVAD implementation,
- the patient is at rest.

These assumptions justify to neglect the autoregulatory mechanisms of cardiovascular pumping such as the systemic baroreflex feedback process and beat to beat myocardium wall strain adaptation based upon the Frank–Starling effect.

#### Feasible Instantaneous Pump Speed Changes

In practice, due to blood inertia, it is impossible to arbitrarily adjust the pump speed. Here we neglect blood and rotor inertia effects and assume that the pump speed can be varied without restrictions. Connected to this, blood is considered as Newtonian fluid and no blood rheology changes are taken into account.

#### Blood Inflow Equals Outflow

In conjunction with the steady-state assumption, we require that the amount of accumulated incoming blood in the LV is equal to the accumulated amount of blood ejected out of the LV over the time horizon $[t_{0},t_{\text {f}}]$. For this purpose we introduce the tolerance parameter $\epsilon _{\text {flow}}>0$ and define (with $Q_{\text {MV}}(t), Q_{\text {AoV}}(t)\ge 0$) the constraint:

6 $$\begin{aligned} \left| \int \limits _{t_{0}}^{t_{\text {f}}} [Q_{\text {MV}}(t) - Q_{\text {AoV}}(t) - Q_{\text {LVAD}}(t)] \ {\text {d}}t \right| \le \epsilon _{\text {flow}}. \ \end{aligned}$$

#### Periodicity of the Heart Cycle

The steady-state assumption implies that it is sufficient to consider only one heart cycle since there are no significant differences between several heart cycles. Thus, in this study we fix the time horizon to the length of one heart cycle. In this way, the steady-state condition translates into a periodicity constraint denoting that the differential state values at the beginning of the heart cycle should be equal to the ones at the end of the cycle. In mathematical terms this results with $\epsilon _{\text {per}}>0$ in

7 $$\begin{aligned} \left| \mathbf {x}_{i}(t_{\text {f}}) - \mathbf {x}_{i}(t_{0}) \right| \le \epsilon _{\text {per}}, \quad {\text {for }} i=1,\ldots ,9. \end{aligned}$$

#### Partial LVAD Support

When using an LVAD in the clinical setting, a distinction is made between full and partial support. While the LV does not contribute to blood ejection through the aortic valve with full support, the aortic valve still opens with partial support because the LV contraction force is still strong enough to pump *partially*. We assume partial support, that is:

8 $$\begin{aligned} \int \limits _{t_{0}}^{t_{\text {f}}} Q_{\text {AoV}}(t) \ {\text {d}}t \ge \epsilon _{\text {partial}}, \ \end{aligned}$$

with $$\epsilon _{\text {partial}}>0$$.

#### Back Flow of Blood from the Aorta in the LV

We want to restrict the back flow from the Aorta in the LV *via* the LVAD. For this, we introduce the tolerance $\epsilon _{\text {back}}>0$ and require

9 $$\begin{aligned} Q_{\text {LVAD}}(t) \ge - \epsilon _{\text {back}}, \quad \text { for } t\in [t_{0},t_{\text {f}}]. \ \end{aligned}$$

#### Adequate Blood Supply

One objective of using an LVAD is to provide sufficient perfusion to the patient’s body. Hence, we seek a pump speed control policy that results in an actual cardiac output that equals approximately a desired and preselected cardiac output $V_{\text {CO}} \in \mathbb {R}_{+}$:

10 $$\begin{aligned} \left| \frac{60}{t_{\text {f}}-t_{0}} \int \limits _{t_{0}}^{t_{\text {f}}} [Q_{\text {AoV}}(t) + Q_{\text {LVAD}}(t)] \ {\text {d}}t - V_{\text {CO}} \right| \le \epsilon _{\text {CO}}, \ \end{aligned}$$

where $\epsilon _{\text {CO}}>0$. We note that the actual cardiac output should not exceed the desired cardiac output up to the tolerance, since this could result in fatigue for the patient.

#### Variable Bounds and Suction Prevention

We require the differential state variables to be in realistic ranges. We denote appropriate lower and upper bounds for the state and control variables with $\mathbf {x}_{\text {lb}}, \mathbf {x}_{\text {ub}} \in \mathbb {R}^{9}$ and $u_{\text {lb}}, u_{\text {ub}} \in \mathbb {R}$. The box constraints read

11 $$\begin{aligned} \mathbf {x}_{\text {lb}} \le \mathbf {x}(t) \le \mathbf {x}_{\text {ub}}, \quad u_{\text {lb}} \le u(t) \le u_{\text {ub}}, \quad \text {for } t\in [t_{0},t_{\text {f}}]. \end{aligned}$$

In this way, we are able to prevent the occurrence of suction, which describes the situation of excessive pumping that may cause a collapse of the ventricle if $P_{\text {LV}}(t)$ is very low.

### Clinical Data and Model Personalization

This study uses data that were obtained retrospectively from the University Hospital Magdeburg, Department of Cardiology, CRT-responder trial,^51^ registered under the Trial Identifier DRKS00011133^3^ and approved by the Institutional Review Board. All patients gave written informed consent. An exemplary subject was selected who involved a dilated LV and suffered from systolic left-sided heart failure. Data collection was performed *via* conductance catheterization for LV pressure measurements and *via* echocardiography for other data. The subject showed in rest a heart frequency of 67 beats per minute with a cardiac output of about 3.5 L/min. Further hemodynamic characteristics of the selected subject are shown in Table 1.

**Table 1:** Measured hemodynamic data for the example subject.

Parameter	End-systolic	End-diastolic
LV volume (mL)	228	281
LV pressure (mmHg)	120	5
PCW pressure (mmHg)	28	14
Aortic pressure (mmHg)	121	53

*PCW* pressure represents a surrogate for LA pressure

We selected a representative cardiac cycle with the duration $h_{\text {cycle}}=0.89$ s and 27 measured data points. We propose to personalize the model *via* a parameter estimation (PE) method. For this, we formulate an optimization problem with the model equations as constraints and a nonlinear regression term as objective that minimizes the difference of model response values to the measured subject data. Here, we minimize the difference between measured LV pressure for selected time points and their corresponding model output values, however, this approach can also be applied to a general measured data set with more differential state types involved. We denote with ${\widehat{P}}_{\text {LV}}(t_{{i}})$ the measured LV pressure at time point $t_{{i}}\in [t_{0},t_{\text {f}}]$. We choose the parameters to be estimated as proposed in Reference 30 with high sensitivity with respect to the LV pressure. These parameters are

$$\begin{aligned} \mathbf {p} = [R_{\text {AVP}}, C_{\text {LV}}, L_{\text {AVP}}, F_{\text {VC}}, F_{\text {AC}}, A_{\text {LV}}, A_{\text {LA}}, k_{\text {RAD}}, S_{\text {D}}]^{\top }. \end{aligned}$$

We bound the parameters to be in a realistic range, i.e., $\mathbf {p}_{\text {lb}}\le \mathbf {p} \le \mathbf {p}_{\text {ub}}$, see Supplemental Material 2 for further details. The selected subject had not (yet) implanted an LVAD, so we set $Q_{\text {LVAD}}(t)$ to zero and neglect the control *u*(*t*) and constraints on $Q_{\text {LVAD}}(t)$ for the PE. The parameter (point) estimation problem is defined as the following optimization problem:

12 $$\begin{aligned}&{\min_{\mathbf{p}}\frac{1}{2}\sum \limits_{i=1}^{n_{\text{m}}} \frac{\left( {\widehat{P}}_{\text{LV}}(t_{i})- P_{\text{LV}}(t_{i}) \right) ^{2}}{\sigma_{i}^{2}} + \phi (\mathbf {p}) } \end{aligned}$$

13 $$\begin{aligned}&{\text{s.t. } {\hspace{0.3cm}}} \dot{\mathbf {x}}(t) = \mathbf {f}(\mathbf {x}(t),\mathbf {p}), \quad \text { for } t\in [t_{0},t_{\text {f}}], \end{aligned}$$

14 $$\begin{aligned}&\mathbf {x}(t_{0}) = \mathbf {x}_{0}, \\&{ {\text {constraints}} (7), (8),(11), } \end{aligned}$$

where $n_{\text {m}}=27$ denotes the number of available measurements, $\mathbf {x}_{0}$ is the initial values, and $\sigma _{i}$ is the standard deviation of the measurement at time $t_{i}$, here set to one. The term $\phi (\mathbf {p})$ allows to incorporate *a priori* information of the parameters, which we here set to zero^4^.

### Optimal Control Problem Formulation

Based on a personalized model, we take interest in an advantageous application of the LVAD for a (possible) patient. An OCP offers the framework to include generic constraints and objective functions. While we have already defined the constraints in “Physiological Assumptions and Constraints” section, for the objective we reuse the multiobjective function from Reference 4. This objective constitutes a compromise function that aims for ventricular unloading and ensures the opening of the aortic valve. A permanent closure of the aortic valve may lead to fusion of the aortic valvular cusps and a resulting thrombus formation.^37^ By ventricular unloading we refer to reducing the hydraulic work that the LV has to perform in order to provide sufficient perfusion. Let $\rho _{1}\in [0,1]$ denote a weighting parameter that facilitates to put one objective more into focus and let $\rho _{2}$ and $\rho _{3}$ denote unit scaling factors, see Supplemental Material 2 for more details. Then, we introduce the objective as

15 $$\begin{aligned} J(\mathbf {x}(\cdot ))&:= \int \limits _{t_{0}}^{t_{\text {f}}} [\rho _{1} \rho _{2} P_{\text {LV}} (t) (Q_{\text {AoV}}(t)+ Q_{\text {LVAD}}(t) \\&\quad -Q_{\text {MV}}(t)) - (1-\rho _{1}) \rho _3 Q_{\text {AoV}}(t) ] \ {\text {d}} t. \end{aligned}$$

The first term accounts for the ventricular unloading, while the second term causes aortic valve opening *via* maximizing the flow through this valve. We consider the following optimization problem, where we minimize the above objective over the differential states $\mathbf {x}(\cdot )$ and the continuous control $u(\cdot )$:

$$\begin{aligned}&\min_{{\mathbf{x}(\cdot ), u(\cdot ) }}J(\mathbf{x}(\cdot)) \\&{\text {s.t. } {\text {model equations }} (5), } \\&{ {\text {inflow equals outflow }} (6), } \\&{ {\text {periodicity of heart cycle }} (7), } \\&{ {\text {partial LVAD support }} (8), } \\&{ {\text {restricted LVAD back flow }} (9), } \\&{ {\text {sufficient perfusion }} (10), } \\&{ {\text {variable bounds }} (11). } \end{aligned}$$

For this optimization problem we investigate three different scenarios regarding the pump speed control.

1. *Constant speed* this represents the usual clinical setting and is expressed by $u(t):=u_{\text {con}}\in [u_{\text {lb}},u_{\text {ub}}]$ for $t\in [t_{0},t_{\text {f}}]$, meaning the pump speed optimization variable is constant over time.
2. *Continuous speed* there are no restrictions on $u(\cdot )$ apart from lower and upper bounds.
3. *Pwc speed* this scenario considers to switch between different constant speed modes, similar as performed in modern devices. For this, we use the indicator function notation
  16 $$\begin{aligned} \chi _{[t_{1},t_{2}]}(t) := {\left\{ \begin{array}{ll} 1, \quad &{} \text {if } t\in [t_{1},t_{2}],\\ 0, \quad &{} \text {else. } \end{array}\right. } \end{aligned}$$
  We assume $u(\cdot )$ to be a step function with three different levels $u_{1},u_{2},u_{3}\in [u_{\text {lb}},u_{\text {ub}}]$:
  17 $$\begin{aligned} u(t) :=&u_{1} \chi _{[t_{0},t_{1})}(t)+u_{2} \chi _{[t_{1},t_{2})}(t)+u_{3} \chi _{[t_{2},t_{3})}(t) \\&\quad + u_{1} \chi _{[t_{3},t_{\text {f}}]}(t), \end{aligned}$$
  where $t_{1},t_{2},t_{3}$ are switching times to be determined^5^. We require minimal time durations for the different speed levels because rapid changes are not feasible in a realistic setting. Let $D_{1},D_{2},D_{3}>0$ denote these so-called minimum dwell times and we introduce the constraints
  18 $$\begin{aligned} t_{1}-t_{0}+t_{\text {f}}-t_{3} \ge D_{1}, \quad t_{2}-t_{1}\ge D_{2}, \quad t_{3}-t_{2}\ge D_{3}. \end{aligned}$$

### Algorithmic Approach

We distinguish between *explicit* and *implicit* switches that result in discontinuous variables for the PE and the OCP. The pump speed control should be in one scenario pwc; however, since we can control when this switch occurs between different speed modes, we call this switch *explicit*. By *implicit* switches we refer to changes of the model equations in $\mathbf {f}$ that happen as soon as the differential states satisfy certain conditions. The valve flows and the contraction force induce such implicit switches. While the valve switches are defined in (2a)–(2b), we specify the contraction force switches in the following.

#### Implicit Switches Through the Contraction Force

As we consider only one heart cycle, the atrial and ventricular contraction takes place once. We assume a physiological order, that is atrial before ventricular contraction followed by a relaxation phase. Initially, let $-S_{\text {D}} \le s(t_{0}) \le S_{\text {D}}$. We further assume the following switching times exist:

19 $$\begin{aligned} t_{{\text {VC}}}&:= \mathop {\text {argmin}}\limits _{t\in (t_{0},t_{\text {f}})}\{ s(t) = - S_{\text {D}}\}, \end{aligned}$$

20 $$\begin{aligned} t_{{\text {R}}}&:= \mathop {\text {argmin}}\limits _{t\in (t_{{\text {VC}}},t_{\text {f}})}\{ s(t)= S_{\text {D}}\}. \end{aligned}$$

Then, the contraction force is defined as

21 $$\begin{aligned} F_{\text {C}}(t) := \left\{ \begin{array}{ll} F_{{\text {AC}}},&{} \quad \text {for } \ t_{0} \le t\le t_{{\text {VC}}} , \\ F_{{\text {VC}}},&{} \quad \text {for } \ t_{{\text {VC}}}< t \le t_{{\text {R}}} , \\ 0, &{} \quad \text {for } \ t_{{\text {R}}} < t \le t_{\text {f}}. \end{array} \right. \end{aligned}$$

#### Dividing the Cardiac Cycle into Phases

**Figure 3:**
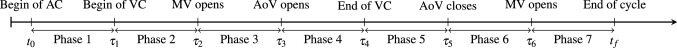
Time course of the assumed active phase sequence with switching events. The switching times $\tau _i$ are variables in the optimization problem.

The periodic switching nature of the cardiac cycle model makes the solving process challenging. We need to identify when switching happens and what the successive active subsystems of $\mathbf {f}$ are. If we combine all possible valve positions and contraction force settings, we get 12 different subsystems. To reduce complexity we assume a specific sequence of active subsystems for the cardiac cycle taking advantage of physiological relationships in the human heart. Thus, we divide the heart cycle into seven phases similar as in Reference 30. Table 2 and Fig. 3 explain the phases of the ordered sequence, where the switching times are denoted with $\tau _{i}$, $i=1,\ldots ,6$.

**Table 2:** Assumed sequence of active phases.

Phase	$F_{\text {C}}$ mode	Mitral valve	Aortic valve
1	AC	Open	Closed
2	VC	Open	Closed
3	VC	Closed	Closed
4	VC	Closed	Open
5	0	Closed	Open
6	0	Closed	Closed
7	0	Open	Closed

For instance, in the first phase the LA contracts, the mitral valve is open and the aortic valve is closed

The modes from Table 2 translate into the following constraints for the optimization problem and for $\mathbf {f}$:

$$\begin{aligned}&`{\text {AC}}`:\, F_{\text {C}}(t)= F_{\text {AC}}, \quad {\text {and}} \quad s(t)> -S_{\text {D}}, \\&`{\text {VC}}`:\, F_{\text {C}}(t)= F_{\text {VC}}, \quad {\text {and}} \quad s(t) < S_{\text {D}},\\&`0`: \, F_{\text {C}}(t)= 0, \\&`{\text {MV open}}`: \, Q_{\text {MV}}(t)=\frac{P_{\text {LA}}(t)- P_{\text {LV}}(t)}{R_{\text {M}}}, \\ &\qquad \qquad {\text {and}} \quad P_{\text {LA}}(t)> P_{\text {LV}}(t),\\&`{\text {MV closed}}`: \, Q_{\text {MV}}(t)= 0, \quad {\text {and}} \quad P_{\text {LA}}(t) \le P_{\text {LV}}(t), \\&`{\text {AoV open}}`:\, Q_{\text {AoV}}(t)=\frac{P_{\text {LV}}(t)- P_{\text {A}}(t)}{R_{\text {AoV}}}, \\ &\qquad \qquad {\text {and}} \quad P_{\text {LV}}(t) > P_{\text {A}}(t),\\&`{\text {AoV closed}}`:\, Q_{\text {AoV}}(t)= 0, \quad {\text {and}} \quad P_{\text {LV}}(t) \le P_{\text {A}}(t). \end{aligned}$$

By fixing the sequence of active subsystems, the PE and OCP transform into multiphase problems,^41^ where only the switching times needs to be determined.

#### Switching Time Optimization

We use switching time optimization^23,41^ to determine the switching times $\tau _{1},\ldots ,\tau _{6}$ so that we can transform the originally discrete optimization problems into continuous ones. The idea of switching time optimization relies on a time transformation $t=({\tilde{t}}_{2}-{\tilde{t}}_{1})\tau$, which exploits that

22 $$\begin{aligned} \dot{\mathbf {x}}(t) = \mathbf {f}(t,\cdot ), \quad t\in [{\tilde{t}}_{1},{\tilde{t}}_{2}], \end{aligned}$$

is equivalent to

23 $$\begin{aligned} \dot{\mathbf {x}}(\tau ) = ({\tilde{t}}_{2}-{\tilde{t}}_{1}) \mathbf {f}(\tau ,\cdot ), \quad \tau \in [0,1]. \end{aligned}$$

With this idea, we reformulate the multiphase model equation:

24 $$\begin{aligned} \dot{\mathbf {x}}(\tau ) = {\left\{ \begin{array}{ll}(\tau _{1} - t_{0}) \mathbf {f}_{1}(\tau ,\cdot ), &{} \quad \text {if } \tau \in [0,1),\\ (\tau _{i} - \tau _{i-1}) \mathbf {f}_{i}(\tau ,\cdot ), &{} \quad \text {if } \tau \in [i-1,i), \text { for } i=2,\ldots ,6,\\ (t_{\text {f}} - \tau _{6}) \mathbf {f}_7(\tau ,\cdot ), &{} \quad \text {if } \tau \in [6,7], \end{array}\right. } \end{aligned}$$

where $\mathbf {f}_{i}$ denotes the model equation for the *i*th phase. We notice that the phase durations enter the equation *via* $(\tau _{i}-\tau _{i-1})$ as continuous variables. At the end of each phase, the switching constraints for the contraction force and the valve flows as described in “Steady-State Situation” section need to be fulfilled at the transformed switching time points up to a tolerance $\epsilon _{\text {sw}}>0$:

25 $$\begin{aligned}&|s(1) + S_{\text {D}} | \le \epsilon _{\text {sw}},\quad |P_{\text {LA}}(2) -P_{\text {LV}}(2)| \le \epsilon _{\text {sw}}, \\&|P_{\text {LV}}(3) -P_{\text {A}}(3)| \le \epsilon _{\text {sw}}, \quad |s(4) - S_{\text {D}} | \le \epsilon _{\text {sw}}, \\&|P_{\text {LV}}(5) -P_{\text {A}}(5)| \le \epsilon _{\text {sw}}, \quad |P_{\text {LA}}(6) -P_{\text {LV}}(6)| \le \epsilon _{\text {sw}}. \end{aligned}$$

This switching time reformulation provides the framework to solve the PE problem and the OCP with constant and continuous pump speed as optimization problem with solely continuous variables. For the pwc pump speed modulation we also need to find the switching times $t_{1},t_{2},t_{3}$ between the three different speed levels as introduced in “Optimal Control Problem Formulation” section. Here, we assume

26 $$\begin{aligned} \tau _{2}< t_{1}<\tau _{3}, \quad \tau _{5}< t_{2}<\tau _{6}, \quad \tau _{6}< t_{3}<t_{\text {f}}, \end{aligned}$$

i.e., the first speed change occurs between mitral valve closing and aortic valve opening, the second between aortic valve closing and mitral valve opening, and the third between mitral valve opening and the end of the heart cycle. Thus, we divide the third, sixth and seventh phase from “Dividing the Cardiac Cycle into Phases” section into two phases each so that in total nine switching times for ten phases need to be determined.

#### Numerical Solution of Optimization Problems

We use direct collocation^59^ to transform the continuous time optimization problems *via* temporal discretization into NLPs. We apply an equidistant discretization grid with time resolution of $\Delta t = 1$ ms. The differential state trajectories are approximated with Radau collocation polynomials^9^ of degree 3. We implemented the optimization problems in python v3.7.5 and used CasADi v3.4.5^5^ to parse the resulting NLP with efficient derivative calculation of Jacobians and Hessians to the solver IPOPT v3.12.3.^63^ For the PE problem we applied the Gauss–Newton method^9^ so that calculation of Hessians is not required.

The lengths of the model phases were extracted from pressure time series and other continuous data and used for initialization of the switching times $\tau _{i}$. These phase durations were fixed for the PE problem and set variable for the OCP. We further initialized the PE problem with variable values based on a simulation with default parameter values, see Supplemental Material 1. The OCP was initialized with simulated variable values obtained with estimated parameters and constant pump speed equal to 8000 rpm. In the OCP, we permitted a deviation of up to 50 ms for the model heart cycle from the subject’s measured cycle length, meaning $t_{\text {f}}\in [0.84,0.94]$. In this way, the constraints (6)–(11) are relaxed in order to enhance robustness of the optimization algorithm.

## Results

### Patient Specification

Solving the PE problem from “Clinical Data and Model Personalization” section resulted in the values

$$\begin{aligned} \mathbf {p}^{*}= [324.2,0.6,20.4,4709,900,42,25,1.35,0.5]^{\top }. \end{aligned}$$

**Figure 4:**
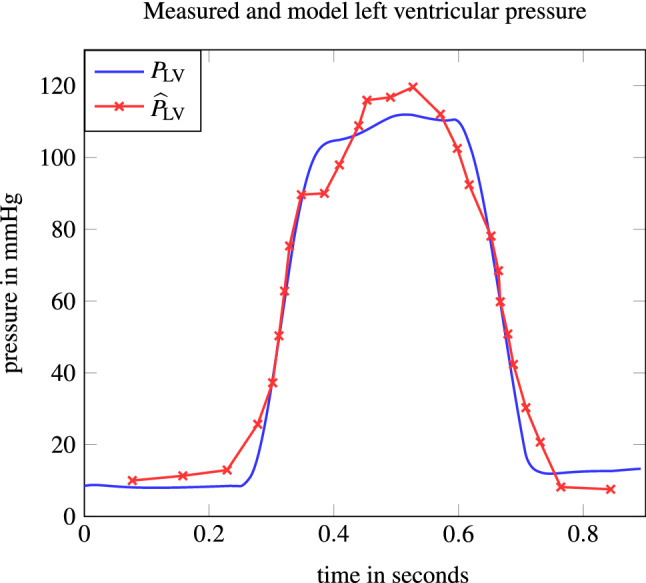
Measured ${\widehat{P}}_{\text {LV}}$ values and resulting $P_{\text {LV}}$ trajectory based on the parameters obtained from solving the PE problem from “Clinical Data and Model Personalization” section.

The result of the switching distance parameter, i.e. $S_{\text {D}}=0.5$, is equivalent to an AVPD of only 10 mm and thus indicates a reduced ventricular function. The situation of heart failure is reflected well by the estimated parameter values. Particularly, the LV compliance is increased, the amplitude of the contraction forces $F_{\text {AC}}$ and $F_{\text {VC}}$ is decreased and the parameter $k_{\text {RAD}}$ accounting for relative contribution of radial pumping is increased. Figure 4 shows the measured data points ${\widehat{P}}_{\text {LV}}$ together with the obtained $P_{\text {LV}}$ from the PE solution.

**Figure 5:**
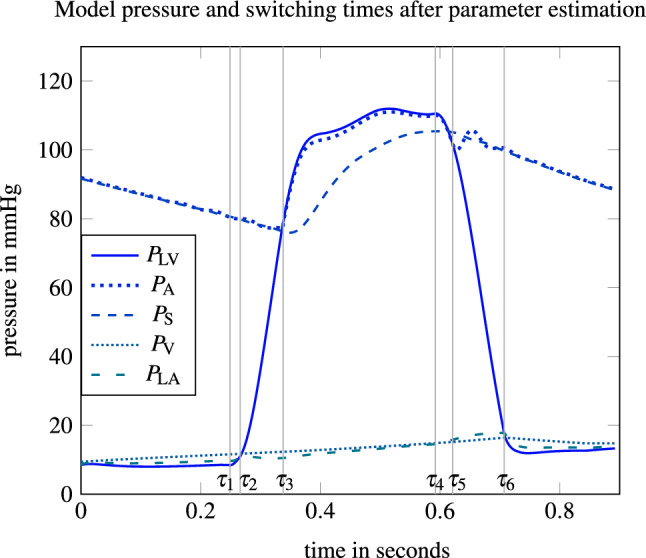
Simulated pressure functions based on the parameters obtained from solving the PE problem from “Clinical Data and Model Personalization” section. The switching times $\tau _{i}$ are depicted with the vertical grey lines.

The model response $P_{\text {LV}}$ reflects the measured data points especially with respect to the duration of ventricular contraction, while its peak is slightly underestimated. The transcripted nonlinear regression problem was solved by IPOPT after 230 s and with an objective value in (12) of 512, which is equivalent to a root-mean-square deviation of 6.16 mmHg. We observed numerical instabilities when solving the PE problem. The convergence of the algorithm seems to depend heavily on the initial solution, which stresses the importance of the proposed initialization from “Numerical Solution of Optimization Problems” section. In addition, we have used mild termination criteria for IPOPT and chose a large tolerance value for the periodicity constraint (7). Figure 5 depicts all model pressure trajectories based on the PE and the six switching times between the different model phases. We observe that the aortic pressure $P_{\text {A}}$ resembles the LV pressure $P_{\text {LV}}$ during ventricular systole and the systemic pressure $P_{\text {S}}$ else, apart from some small oscillations. Likewise, $P_{\text {LA}}$ and $P_{\text {V}}$ represent similarly high pressures, although $P_{\text {LA}}$ adopts to $P_{\text {LV}}$ depending on the mitral valve opening. While the left atrial pressure range is between 10 and 20 mmHg and, thus, appears realistically,^57^ the missing increase of $P_{\text {LA}}$ at the end of diastole seems to indicate that the left atrial function is partly inappropriately modeled.

### Pump Control Policies

**Figure 6:**
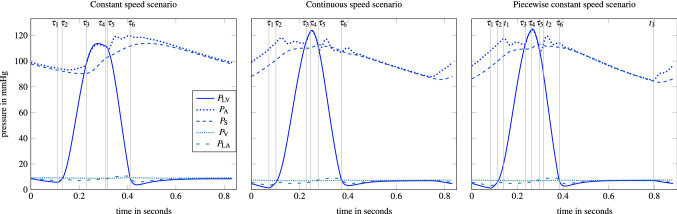
Calculated pressure differential states for the OCP solutions with different pump speed scenarios. The switching times $\tau _{i}$ and pwc speed changing times $t_{i}$ are depicted with the vertical grey lines.

We solved the OCP according to the proposed algorithm from “Algorithmic Approach” section. The applied parameters for the objective and constraints such as the tolerance parameters $\epsilon _{\cdot }$, the dwell times $D_{\cdot }$, and lower and upper bounds on variables are listed in Supplemental Material 2. Figure 6 shows the pressure functions for the three pump speed scenarios. The outcomes of the continuous and pwc scenario are very similar. They show an elevated peak of $P_{\text {LV}}$ compared to the parameter estimated solution from Fig. 5. While the rise and fall of the pressure profiles before and after the ventricular contraction is significantly steeper than with the parameter estimated solution, its duration, i.e. $\tau _{4}-\tau _{1}$, is in a similar range due to an enforced minimum dwell time of 0.2 s for the ventricular contraction. Very low values occur for $P_{\text {LV}}$ directly before $\tau _{1}$, however, they are still above the threshold for suction. We notice that the cycle duration for the pwc scenario is 0.88 s and slightly longer than for the other two scenarios with a duration of about 0.84 s, as we allow a slight deviation of 50 ms from the standard cycle length. The constant speed scenario results in $u(\cdot )\equiv 10,036$ rpm and involves also an increased aortic and ventricular pressure compared to the parameter estimated solution, though their peaks are significantly lower compared with the continuous and pwc speed scenarios.

**Figure 7:**
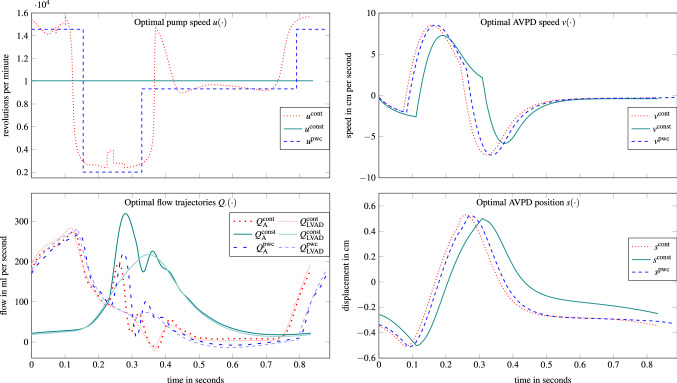
Calculated pump speed and differential states for the OCP solutions. The superscripts *cont, const,* and *pwc* abbreviate *continuous, constant* and *piecewise constant* rotor speed.

Figure 7 illustrates the different optimal pump speed profiles and the according results for the flows, AVPD speed, and AVPD position. We observe that the continuous speed profile provides counterpulsative pump support and the pwc speed profile approximates this profile. Due to this similar pump speed behavior, the optimal differential state trajectories resemble each other. The main portion of the flows through the LVAD and the aorta appears for constant pump speed during ventricular contraction. In contrast, with continuous or pwc speed, large flow values occur already during atrial contraction followed by a peak during ventricular contraction, which accounts for the remaining physiological contraction force. The flow for the continuous pump speed appears to be slightly negative around $t=0.4$ since we relaxed the tolerance parameter in (9) for achieving numerical convergence. The upper right panel shows that the larger amplitudes of the pumping speed for the continuous and pwc scenario compared with constant speed translate into faster AVP movements. The more pronounced ventricular unloading in the continuous and pwc scenario also leads to the AVPD speed peak and the switching distance (lower right panel) being reached earlier than in the constant speed scenario. Since the parameter $S_{\text {D}}$ is fixed, there is no difference in the AVPD distance realized between the scenarios. However, the AVP in the continuous and pwc scenarios returns to its starting position earlier than in the constant speed scenario, which can again be explained by more pronounced unloading.

**Figure 8:**
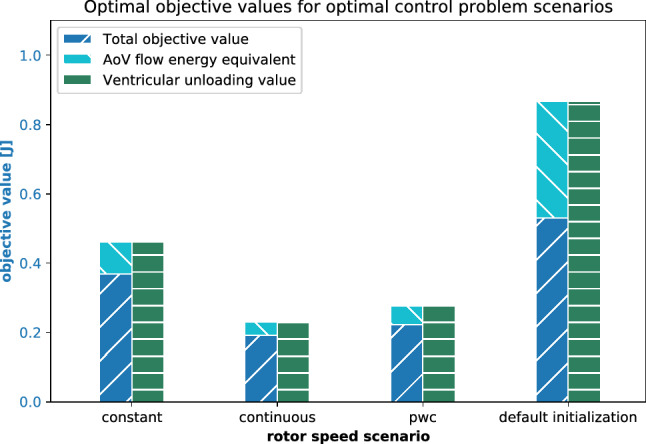
Comparison of constructed optimal objective values for the OCP from “Optimal Control Problem Formulation” section. The objective values for the default initialization are obtained with $u(\cdot )\equiv 8000\,\text {rpm}$. Notice that the total objective value results from subtracting the aortic valve flow value from the ventricular unloading, as defined in (15). The objective value decreases from 0.531 after initialization to 0.368 with constant speed. Continuous and pwc speed modulation construct even lower objective values, which amount to 0.191 and 0.222, respectively.

Figure 8 summarizes the objective values and runtimes for the OCP solutions. Clearly, the obtained objective value with the pwc speed profile is only slightly larger than the one calculated with continuous pump speed, while the objective value with constant speed is not competitive.

## Discussion

The root-mean-square error between ${\widehat{P}}_{\text {LV}}$ and $P_{\text {LV}}$ is about twice as large as the one constructed *via* a trial method in Reference 30. However, the latter study did not take into account the constraints (7), (8), and (11) during the data fitting procedure. The performed PE should be discussed critically with respect to overfitting since we optimized nine parameters with available measurements for only one differential state. To this end, we calculated the relative standard deviation (SD) values based on the Fisher-information matrix as defined in Reference 33, which represents a common criteria for evaluating the quality of the results of a PE. The SD values with respect to the optimal parameter vector $\mathbf {p}^{*}$ are

$$\begin{aligned} \% SD (\mathbf {p}^{*})&= [ 42.3, 24.5, 40.4, 35.5, 354.2, 34.6, 267.7,49.1, 26.2 ]^{\top }. \end{aligned}$$

The calculated SD values are mostly quite low and thus indicate a robust quality of the obtained estimation. In contrast, the values for $F_{\text {AC}}$ and $A_{\text {LA}}$ with about 354 and 268% are quite large and we postulate that they have a minor impact on $P_{\text {LV}}$. Future work should focus on sensitivity analysis for choosing the right parameters to be estimated.

The increased peak of the LV pressure function after using pumping assist is consistent with typical partial LVAD support^44^ The causality can be explained as follows: The LVAD delivers continuously blood to the aorta, increasing the aortic pressure. Therefore the LV pressure must be elevated for the aortic valve to open. This effect is more pronounced in the scenarios with continuous and pwc pump speed, which can be categorized as counterpulsative policies and thus facilitate the aortic pressure to oscillate less.

The counterpulsative pumping involves high rotational speeds during diastole and low speeds in systole and provides minimization of the objective function in two ways. First, strong LVAD pumping before the ejection phase causes a substantial amount of blood to already pass from the ventricle into the aorta, thus unloading the ventricle. Second, low rotational velocities during systole cause a lower aortic pressure and therefore a large aortic valve flow. Our finding that a counterpulsative pumping strategy minimizes the given objective function is in accordance with Reference 3, where the same objective function was applied.

In the continuous scenario, an increased rotational speed at the beginning of diastole is also noticeable, which is necessary to prevent and limit LVAD backflow [Constraint (9)].

After applying the LVAD support, we observed shortened atrial contraction phases, which is equivalent to shortened ventricular filling phases. We interpret this behavior as a result of maximizing the ventricular unloading. This implies the LA pumps against less resistance and reaches the maximum contraction state more quickly, which is represented by theAVP reaching the switching distance $-S_{\text {D}}$. In this way, the AVPD model can realistically capture interactions of an LVAD and the cardiac system, as elaborated in References 15, 62. The continuous and pwc pump speed scenarios have more degrees of freedom compared with the constant speed scenario and thus improve the diastolic function even more as depicted by the shortened atrial contraction phases (0.111, 0.073, and 0.083 s in constant, continuous, and pwc scenarios, respectively).

With regard to the constraints, it should be noted that some conditions significantly shape the optimisation result. These include sufficient blood perfusion (10), periodicity constraint (7), and the blood flow balance constraint (6). In contrast, some of the variable bounds and the backflow condition (9) turned out to be often not active in the optimization process and can be (partly) neglected.

### Connection to Clinical Application

LVADs work in an online environment where the system state can change rapidly, especially the heart rate and blood volume shift. Currently, there are no sensors available that provide long-term measure signals of the presented differential states,^12^ including $Q_{\text {AoV}}$ and $P_{\text {LV}}$ for the objective function. Despite these aspects and our restricting assumptions (see next subsection), we claim that optimized speed profiles from offline computations may provide superior performance (see Fig. 8) and could be considered in the following way.

1. The optimal control framework can be used to benchmark a whole range of speed profiles, in particular modern pwc speed profiles, that result from different objective functions, models, and constraints.
2. These evaluations can be carried out on a patient-specific basis. As a proof of concept, we demonstrated for one patient that the cardiovascular model can be efficiently altered to represent the patient’s LV pressure function. This approach can be extended to include time series measurements of the aortic and LA pressure (*via* PCW pressure and conductance catheterization), the flows at the mitral and aortic valve, and the AVP speed and displacement (all *via* Doppler echocardiography). Overall, at least five differential states could be used for model personalization. The data used so far were measured invasively. In contrast, in routine clinical investigations, echocardiography can be used to measure and use non-invasive data concerning approximated pressure–volume (PV) loops and time series of valve flows.
3. Offline computations of an optimal speed profile for different situations, e.g. rest, exercise or rhythm disturbances such as atrial fibrillation, could be done beforehand and used in an online setting assuming information about the system status is available.
4. The presented algorithmic idea can be extended to model predictive control. In particular, the possibility to incorporate minimum dwell time constraints to avoid rapid speed changes paves the way for a realistic extension to the online setting. Here, computational improvements on the algorithm and the implementation would be necessary to cope with the real-time setting.

### Limitations

#### Simplified Model

We applied a lumped model that simplifies the heart and the cardiovascular system by neglecting the right heart, the pulmonary system, valve regurgitation, and spatial interactions between compartments. Dilated heart failure is very commonly associated with valve regurgitation so that our assumption to neglect it should be seen as critical. The model can be easily extended to capture valve regurgitation by replacing the zero value in the flow equations (2a) and (2b) with a backflow value that corresponds to the valve insufficiency. According to the Frank–Starling law, the passive LV compliance $C_{\text {LV}}$ is set to be constant but may change instantaneously. The way we model the LVAD and its interaction with the heart is also highly simplified. Moreover, neurological feedback processes such as the baroreflex are not captured.

#### Assumptions

In reality, the heart rate and thus the duration of the cycle is very variable, especially through exertion or sport. In most cases of patients requiring the VAD therapy the stable heart rhythm is a scarce phenomenon. The rhythm disturbances are rather the dominant pattern in the individuals suffering from heart failure. Therefore the steady state assumption must be viewed critically. Furthermore, we neglect rotor and blood inertia so that the rotor speed can be controlled arbitrarily. Nevertheless, our framework may be good as a general starting point for the twofold development as part of future work. First we may be able to base the optimization process on critical parameters with respect to clinical availability. Second the early detection of atrial fibrillation as the most common rhythm disturbance in heart failure could be implemented to switch the working regime of the pump into different mode.^49^

#### Measured Data

We included in this study only one subject and measured data for only one differential state. Future work shall address numerical tests with several patients, with additional measured states, with multiple cycles for model personalization, and with additional beats as validation data set.

#### Control Approach

Other measures of ventricular work such as the PV area could be applied for the objective function. Our numerical result that counterpulsative speed modulation maximizes ventricular unloading but neglects pulsatility are consistent with a bovine study.^56^ In that study, copulsive pumping and asynchronous pumping relative to the cardiac cycle were beneficial for pulsatility. In the future, the presented algorithm could be used to find optimal pump speed trajectories related to pulsatility, ideally over several cardiac cycles. This study assumes a canonical order of the active phases with respect to the valves and the contraction force. This order might not always be true in practice so that future work should consider optimization with implicit switches, but without fixing the order of active phases. Besides, we optimize over one heart cycle, whereas the pulsatility speed mode profiles of some LVADs such as HeartMate 3^TM^ last for more than one heart cycle. Regarding the numerical results, we note that we found local optima by using the solver IPOPT, as finding global optima for OCPs can be very laborious.^19^

#### Pump Flow Rates

The realistic range of LVAD pump flow rate is between 2 and 10 L/min, where the lower bound is due to the risk of thrombosis. The computed pump flow rates fall below or exceed this range on some time points but are on average over the whole heart cycle in the realistic range. Moreover, the almost instantaneous flow rate changes from almost 0 up to 18 L/min will not happen in practice. This study permits a 800% increase from the lowest pump speed set point, whereas currently available LVADs allow an approximate increase of 200%.

### Switched Systems Framework Applicability

The developed multiphase algorithm is applicable to other models and settings. For example, the OCP can also be interpreted on a cardiac model with time-varying elastance function as a switched system, with the valves still representing the implicit switches and changes of the constant pump speed representing “controllable” switches. Analogously, the framework can be beneficial for PE of cardiac models without LVAD application, but with different scope, e.g. cardiac resynchronization therapy.

The presented algorithmic framework is suitable for longer time periods than a single cardiac cycle, as the switching sequence can be extended to the subsequent heart beats.

There are similar devices to an LVAD available or under development for which an OCP could be solved efficiently with the switched systems framework. For instance, total artificial hearts such as RealHeart^58^ and Carmat^55^ or intra-aortic blood pumps^22^ involve also discrete system changes, induced by piston pumps (RealHeart), controlled valves (Carmat) or pulsatility rotor speed modes (intra-aortic blood pump). Finally, the next generation LVADs may include more advanced control features that can lead to different control modes to switch on/off. The TORVAD device^26^ falls into this category and works with two magnetic pistons within a torus generating pulsatile flow.

### Concluding Remarks

We have proposed a novel switched systems algorithm for the optimal control of LVADs that provides the opportunity to calculate optimal constant, pwc, or continuous pump speed profiles. As a proof of concept, we showed that this algorithm can be used to adapt a cardiovascular model to patient specific data and to benchmark simulations of personalized LVAD control policies. The importance of achieving hemodynamic optimization in LVAD patients is highlighted by a significantly lower rate of hospital readmissions,^32^ and could benefit from in silico analysis such as the presented speed profile evaluations. Moreover, we have demonstrated realistic simulations of a model that is based on AVPD instead of using the widespread time-varying elastance model and examined thereby the heart to LVAD interactions. Future work may test the algorithm on more patient data, more realistic conditions such as exercise or rhythm disturbance, and with model extensions. The proposed algorithm could be also beneficial for the evaluation of pulsatile speed modulation modes of modern devices such as HeartMate 3^TM^ or HeartWare HVAD^TM^.

## Supplementary Information

Below is the link to the electronic supplementary material.

Supplementary file1 (PDF 125 kb)

## Footnotes

1 We neglect valve regurgitation and set the back flow to zero. We discuss this assumption in “Limitations” section.

2 Alternatively, myocardial infarction is a common case related to LVAD patients; however, it is challenging to represent scars adequately with a zero-dimensional lumped model.

3 https://www.drks.de/drks_web/navigate.do?navigationId=trial.HTML&TRIAL_ID=DRKS00011133.

4 Future work should consider a priori information in the form of $\phi (\mathbf {p})=\epsilon \Vert \mathbf {p}-\bar{\mathbf {p}} \Vert ^{2}$ instead of imposing lower and upper bounds on $\mathbf {p}$.

5 In fact, we can drop $\chi _{[t_{3},t_{\text {f}}]}(t)$ since $\mathbf {x}(0)$ is free. However, the algorithmic idea in the next section exploits a fixed sequence of active system phases so that we keep this term.
