# Supplementary material for: An Intra-Cycle Optimal Control Framework for Ventricular Assist Devices Based on Atrioventricular Plane Displacement Modeling
Source: Ann Biomed Eng. 2021 Sep 21;49(12):3508–23. doi: 10.1007/s10439-021-02848-2 (PMC8671277; doi:10.1007/s10439-021-02848-2)
Supplement: Supplementary file 1 — Supplementary file1 (PDF 125 kb) [file 10439_2021_2848_MOESM1_ESM.pdf]

Supplemental Material

1 Model parameter values

The resistance and inertance of the LVAD is captured by the parameters R_{LVAD} and L_{LVAD}. We use the values from [53] as shown in Table Table S1 and given by

$$L_{\mathrm{LVAD}} = L_i + L_o + 0.02177,$$
$$R_{\mathrm{LVAD}} = R_i + R_o + 0.1707.$$

We altered the model from [30] to the situation of left heart failure by increasing the left atrial and ventricular compliance, increasing the systemic resistance, decreasing the length of AVPD, increasing the cross-section of LA and LV in order to account for a dilated heart, and decreasing the contraction force F_C. The specific parameter values are defined in Table Table S1.

Table S1 Default parameter values for the cardiovascular, circulatory system, and LVAD model.

Parameter	Description	Value
C_{LA}	Left atrial compliance	0.6 ml/mmHg
C_{LV}	Left ventricular compliance	0.45 ml/mmHg
C_{A}	Aortic compliance	0.08 ml/mmHg
C_{S}	Systemic arterial compliance	1.2 ml/mmHg
C_{V}	Venous compliance	50 ml/mmHg
R_{M}	Mitral valve resistance	0.005 mmHg s/ml
R_{AoV}	Aortic valve resistance	0.005 mmHg s/ml
R_{S}	Systemic arterial resistance	1.1 mmHg s/ml
R_{C}	Characteristic resistance	0.05 mmHg s/ml
R_{i}	LVAD inlet resistance	0.0677 mmHg s/ml
R_{o}	LVAD outlet resistance	0.0677 mmHg s/ml
R_{AVP}	Damping of AVP	300 mmHg cm s
L_{S}	Arterial inertance	0.001 mmHg s^2/ml
L_{i}	Inlet inertance of LVAD	0.0127 mmHg s^2/ml
L_{o}	Outlet inertance of LVAD	0.0127 mmHg s^2/ml
L_{AVP}	Inertia of AVP	30 mmHg cm s^2
β	Pump-to-pressure coefficient	-9.9025e-7
A_{LA}	Left atrial cross-section	25 cm^2
A_{LV}	Left ventricular cross-section	50 cm^2
F_{AC}	Left atrial contraction force	1000 mmHg cm^2
F_{VC}	LV contraction force	5000 mmHg cm^2
k_{RAD}	Radial function coefficient	1.2
S_{D}	Switching threshold for AVP	0.4 cm

2 Optimization parameter values

The following lower and upper bounds for the variables of the optimization problems were applied.

$$\mathbf{p} = [R_{\mathrm{AVP}}, C_{\mathrm{LV}}, L_{\mathrm{AVP}}, F_{\mathrm{VC}}, F_{\mathrm{AC}}, A_{\mathrm{LV}}, A_{\mathrm{LA}}, k_{\mathrm{RAD}}, S_D]^\top,$$
$$\mathbf{p}_{\mathrm{lb}} = [100, 0.45, 15, 4500, 900, 30, 15, 0.5, 0.2]^\top,$$
$$\mathbf{p}_{\mathrm{ub}} = [600, 5, 60, 7000, 1500, 42, 25, 1.35, 0.6]^\top,$$
$$\mathbf{x} = [P_{\mathrm{LA}}, P_{\mathrm{LV}}, P_{\mathrm{A}}, P_{\mathrm{S}}, P_{\mathrm{V}}, Q_{\mathrm{A}}, Q_{\mathrm{LVAD}}, v, s]^\top,$$
$$\mathbf{x}_{\mathrm{lb}} = [0, 0, 40, 0, 0, -100, -60, -15, -1.2]^\top,$$
$$\mathbf{x}_{\mathrm{ub}} = [60, 150, 160, 160, 150, 1000, 300, 15, 1.2]^\top,$$
$$\mathbf{u}_{\mathrm{lb}} = 2000, \quad \mathbf{u}_{\mathrm{ub}} = 16000.$$

We used as initial differential state value for the parameter estimation problem:

$$\mathbf{x}_0 = [8, 8, 90, 90, 8, 10, 0, 0, -0.2]^\top.$$

Furthermore, the following parameters for constraints were applied:

$$\varepsilon_{\mathrm{flow}} = 10 \text{ ml/s}, \quad \varepsilon_{\mathrm{back}} = -40 \text{ ml/s}, \quad \varepsilon_{\mathrm{CO}} = 80 \text{ ml},$$
$$V_{\mathrm{CO}} = 5000 \text{ ml}, \quad \varepsilon_{\mathrm{sw}} = 0.001, \quad \varepsilon_{\mathrm{per}} = 0.09,$$
$$\varepsilon_{\mathrm{partial}} = 0.01 \text{ ml}.$$

The minimum dwell times for the pwc pump speed levels were

$$D_1 = 0.1s, \quad D_2 = 0.1s, \quad D_3 = 0.1s.$$

We also enforced upper bounds τ_{ub} on the seven phase durations to be

$$\tau_{\mathrm{ub}} = [0.3, 0.2, 0.2, 0.2, 0.2, 0.3, 0.5]^\top \text{seconds}.$$

The objective parameters are

$$\rho_1 = 0.5, \quad \rho_2 = 0.000133\text{J / (mmHg ml)}, \quad \rho_3 = 0.01\text{J s/ml}.$$

We used the following `IPOPT` setting: *'tol': 1e-6, 'constr_viol_tol': 1e-6, 'compl_inf_tol': 1e-6, 'dual_inf_tol': 1e-6.*
